# A prognostic model for colorectal cancer based on CEA and a 48-multiplex serum biomarker panel

**DOI:** 10.1038/s41598-020-80785-1

**Published:** 2021-02-22

**Authors:** Kajsa Björkman, Sirpa Jalkanen, Marko Salmi, Harri Mustonen, Tuomas Kaprio, Henna Kekki, Kim Pettersson, Camilla Böckelman, Caj Haglund

**Affiliations:** 1 Research Programs Unit, Translational Cancer Medicine, University of Helsinki, Meilahti Hospital, Haartmaninkatu 4, PO Box 340, 00029 HUS Helsinki, Finland; 2 MediCity Research Laboratory and Institute of Biomedicine, University of Turku, Turku, Finland; 3 Department of Biochemistry, University of Turku, Turku, Finland; 4 Department of Surgery, University of Helsinki and Helsinki University Hospital, Helsinki, Finland

**Keywords:** Gastrointestinal cancer, Colorectal cancer, Immunochemistry, Cancer, Computational biology and bioinformatics, Biomarkers, Gastrointestinal cancer, Tumour immunology

## Abstract

Mortality in colorectal cancer (CRC) remains high, resulting in 860,000 deaths annually. Carcinoembryonic antigen is widely used in clinics for CRC patient follow-up, despite carrying a limited prognostic value. Thus, an obvious need exists for multivariate prognostic models. We analyzed 48 biomarkers using a multiplex immunoassay panel in preoperative serum samples from 328 CRC patients who underwent surgery at Helsinki University Hospital between 1998 and 2003. We performed a multivariate prognostic forward-stepping background model based on basic clinicopathological data, and a multivariate machine-learned prognostic model based on clinicopathological data and biomarker variables, calculating the disease-free survival using the value of importance score. From the 48 analyzed biomarkers, only IL-8 emerged as a significant prognostic factor for CRC patients in univariate analysis (HR 4.88; 95% CI 2.00–11.92; *p* = 0.024) after correcting for multiple comparisons. We also developed a multivariate model based on all 48 biomarkers using a random survival forest analysis. Variable selection based on a minimal depth and the value of importance yielded two tentative candidate CRC prognostic markers: IL-2Ra and IL-8. A multivariate prognostic model using machine-learning technologies improves the prognostic assessment of survival among surgically treated CRC patients.

## Introduction

Colorectal cancer (CRC) leads to 860,000 deaths worldwide annually^1^. Thus, CRC represents a global health hazard with an enormous impact on global health and welfare. Furthermore, CRC becomes increasingly common as countries further develop given that rates of CRC increase as living standards improve^1^.

Several clinicopathological methods have been developed to classify CRC and predict disease progression. TNM staging takes into account the microanatomy, nodule and organ metastases, while the Dukes modified classification–previously in wide use–is based on these corresponding factors^2^. Histopathological classification of CRC tumors relies on the tumor grade^2^. If the patient’s health allows it, surgery remains the primary treatment for CRC. Furthermore, patients with distal rectal cancer receive neoadjuvant radio- or chemoradiotherapy. Depending upon the tumor staging and location, high-risk patients typically receive adjuvant therapy^3^.

Carcinoembryonic antigen (CEA) is routinely recommended during the postoperative follow-up of CRC patients^3^, and is also used for prognostic evaluation given its superiority to other standalone prognostic biomarkers. However, alone CEA carries a limited accuracy as a prognostic factor^3,4^. Some predictive models using computational intelligence methods have been developed, although we lack a consensus model for CRC prognostics^5,6^. Therefore, trials to develop multivariate background models are needed^7^. Some researchers have assumed that models using artificial intelligence (AI) would provide a superior accuracy compared to conventional methods^8^.

In this study, we aimed to develop a multivariate prognostic CRC model including biomarkers combined with clinical background data. Here, we compare the performance of this model—that is, the study model—to a model based solely on basic conventional clinicopathological background data plus CEA. We hypothesized that the study model would perform better than the background model.

## Patients and methods

### Study design

We analyzed preoperative serum samples from CRC patients undergoing elective surgery, excluding patients with previous malignancies or synchronous tumors. Each tumor was histologically verified as an adenocarcinoma by a pathologist and we used the American Joint Committee on Cancer’s (AJCC) seventh edition of tumor staging to assess TNM. Serum samples were then analyzed using multiplex assays. Survival analyses and the multivariate prognostic model were designed in preparation of the serum sample results.

### Patients

The cohort included 328 CRC patients who underwent elective surgery between 1998 and 2003 at Helsinki University Hospital (Supplementary Table 1). The Finnish Population Register Center provided the survival data needed to compute the survival statistics, and Statistics Finland provided cause-of-death information. From these data, disease-specific survival (DSS) could be calculated, and we updated survival data in September 2019. Patient information, samples and data were handled and stored in accordance with the Declaration of Helsinki and other local regulations.

### Serum samples

Serum samples were taken before bowel preparation and before surgery (median 1 day, range 0–30 days), aliquoted and stored at – 80 °C until assayed in 2018.

### Protein profiling

Unthawed serum samples were used for the measurement of 48 cytokines, chemokines and growth factors using Bio-Rad’s premixed Bio-Plex Pro Human Cytokine 27-plex Assay (catalog no. M500KCAF0Y) and 21-plex Assay (catalog no. MF0005KMII) kits on Bio-Rad’s Bio-Plex 200 System (Supplementary Table 2). All assays were prepared according to the manufacturer’s instructions. However, we used one-half the recommended concentration levels for the number of detection antibodies, beads and the streptavidin–phycoerythrin conjugate. This approach was previously validated^9,10^.

The CEA concentrations were measured from the same serum samples using an enzyme-linked immunosorbent assay (ELISA) (Fujirebio Diagnostics AB, Gothenburg, Sweden).

### Statistics

The endpoint for the prognostic evaluation was disease-specific survival (DSS), defined as the time from surgery until death from CRC. We used biomarkers as continuous variables in the univariate Cox regression analyses, all of which were analyzed using the false discovery rate (FDR) for the multiple-test correction^11^. We chose background characteristics consisting of patient age, tumor location, stage and gender for the multivariate survival analysis using the Cox regression model. We also calculated time-dependent receiver operating characteristic (ROC) curves and the area under the curves (AUCs) using the TimeROC package in R, and the integrated AUC over time from 6 to 60 months.

In addition, we tested the distributions for the continuous variables using the Mann–Whitney *U* test and the Kruskal–Wallis test. Survival time was estimated using the Kaplan–Meier method using dichotomized biomarker levels.

For variable selection to identify tentative prognostic markers for survival in CRC, we also used random survival forest modeling. We applied a terminal node size of 19 with 5000 trees, and sampling was completed with replacement and applied the gradient-based brier score-splitting rule. Random survival forest analysis was performed using the R packages randomForestSRC (https://github.com/kogalur/randomForestSRC) and ggRandomForests (https://github.com/ehrlinger/ggRandomForests).

We used two-tailed *p* values and considered *p* < 0.05 statistically significant. Statistical evaluations were calculated using IBM’s statistical software (IBM SPSS Statistics Version 25, International Business Machines Corp., NY, USA) and R version 3.4.3 (Foundation for Statistical Computing, Vienna, Austria).

### Ethical approval

The Ethics Committee at the University of Helsinki approved the study protocol (226/E6/2006, extension 17.4.2013). The National Supervisory Authority of Health and Welfare approved the retrospective study (Valvira Dnro 10041/06.01.03.01/2012). Patients provided their written informed consent upon inclusion in the study. Patient information, samples and data were handled and stored in accordance with the Declaration of Helsinki and other local regulations.

## Results

The mean age of patients was 66.5 years (range 31.7–92.7), and 157 (47.9%) patients were women. Among all patients, 56 had stage I, 100 stage II, 113 stage III and 59 stage IV CRC. Right-sided disease was seen in 90 (27.4%) patients and colon cancer in 153 (46.6%; Supplementary Table 1).

### Univariate survival analysis

Serum values were calculated as continuous values and the Cox regression analyses were performed at logarithm base 10 for the biomarker serum values. Among 48 analyzed biomarkers, six biomarkers resulted in *p* < 0.05: interleukin 6 (IL-6), interleukin 8 (IL-8), interleukin 2 receptor alpha chain (IL-2Rα), cutaneous T-cell attracting chemokine (CTACK), macrophage migration inhibitory factor (MIF) and stromal cell-derived factor 1 alpha (SDF-1α). From these, one biomarker, IL-8, resulted in *p* < 0.05 following FDR correction (hazard ratio [HR] 4.88; 95% confidence interval [95% CI] 2.00–11.92; *p* = 0.024; Table 1). Patients with high levels of IL-8 exhibited a poor prognosis.

**Table 1:** Univariate analysis of biomarkers analyzed using the Bio-Rad’s premixed Bio-Plex Pro Human Cytokine 27-plex Assay and 21-plex Assay.

Biomarker	Median (pg/ml)	IQR (pg/mL)	HR*	95% CI*	*p* value*	FDR-corrected *p* value*
IL-1b	7.66	6.11–9.55	1.16	0.441–3.04	0.768	0.959
IL-1ra	84.4	62.3–119	1.13	0.555–2.29	0.740	0.959
IL-2	0.86	0.770–5.62	1.08	0.847–1.37	0.538	0.959
IL-4	17.7	15.2–22.2	0.750	0.285–1.97	0.560	0.959
IL-5	8.31	3.54–17.9	0.990	0.726–1.35	0.947	0.966
**IL-6**	**13.8**	**8.81–23.7**	**1.64**	**1.082–2.50**	**0.020**	0.250
IL-7	21.8	13.1–29.7	0.917	0.560–1.50	0.730	0.959
**IL-8**	**43.2**	**32.8–61.4**	**4.88**	**2.00–11.92**	**< 0.001**	**0.024**
IL-9	320	265–389	0.978	0.432–2.22	0.957	0.966
IL-10	12.0	6.94–18.5	1.17	0.782–1.74	0.451	0.959
IL-12p70	42.2	30.2–62.3	1.41	0.812–2.44	0.223	0.666
IL-13	5.32	3.46–8.99	1.17	0.719–1.91	0.521	0.959
IL-15	0.84	0.662–1.38	1.06	0.821–1.37	0.659	0.959
IL-17	141	107–180	1.62	0.541–4.86	0.389	0.959
Eotaxin	165	126–222	0.875	0.448–1.71	0.696	0.959
FGF basic	164	122–204	0.883	0.306–2.55	0.819	0.959
G-CSF	128	96.8–171	1.03	0.408–2.62	0.945	0.966
GM-CSF	2.64	0.978–23.4	1.03	0.831–1.28	0.770	0.959
IFN-g	88.4	70.0–111	1.07	0.449–2.57	0.874	0.966
IP-10	1690	1120–2440	1.02	0.549–1.89	0.955	0.966
MCP-1	28.6	20.6–40.4	1.07	0.667–1.70	0.789	0.959
MIP-1a	6.10	4.85–7.81	1.29	0.437–3.83	0.642	0.959
PDGF-bb	6150	4430–8150	0.928	0.507–1.70	0.809	0.959
MIP-1b	274	176–398	0.806	0.441–1.47	0.482	0.959
RANTES	43,700	5980–90,000	0.930	0.707–1.22	0.608	0.959
TNF alpha	203	171–241	1.04	0.425–2.56	0.927	0.966
VEGF	94.8	60.2–139	1.18	0.597–2.35	0.628	0.959
IL-1a	0.417	0.394–1.44	1.11	0.698–1.75	0.668	0.959
**IL-2Ra**	**130**	**93.70–184**	**2.35**	**1.09–5.09**	**0.030**	0.271
IL-3	46.5	15.4–92.2	1.18	0.892–1.56	0.246	0.666
IL12p40	4.84	4.46–6.74	0.951	0.725–1.25	0.717	0.959
IL-16	116	76.7–172	1.62	0.866–3.04	0.131	0.662
IL-18	92.0	66.3–128	1.69	0.731–3.91	0.219	0.666
**CTACK**	**973**	**739–1240**	**2.92**	**1.09–7.86**	**0.034**	0.271
GROa	158	114–225	1.66	0.769–3.57	0.197	0.666
HGF	707	500–958	1.29	0.599–2.76	0.520	0.959
IFN-a2	15.4	3.22–31.0	1.23	0.865–1.75	0.248	0.666
LIF	8.71	1.92–20.8	1.12	0.808–1.55	0.500	0.959
MCP-3	2.31	1.72–8.33	0.994	0.757–1.31	0.966	0.966
MCSF	17.6	12.1–27.1	1.52	0.856–2.71	0.152	0.663
**MIF**	**1380**	**795–2190**	**1.97**	**1.11–3.50**	**0.021**	0.250
MIG	259	165–452	1.75	0.985–3.12	0.056	0.359
b-NGF	3.05	1.03–6.89	1.27	0.861–1.87	0.229	0.666
SCF	157	127–198	1.73	0.679–4.42	0.250	0.666
SCGF-b	15,700	11,500–21,000	1.15	0.570–2.33	0.693	0.959
**SDF-1a**	**92.9**	**74.3–108**	**6.26**	**1.36–28.9**	**0.019**	0.250
TNF b	6.81	3.91–10.3	1.46	0.885–2.43	0.138	0.662
TRAIL	141	97.9–208	0.691	0.470–1.02	0.060	0.359

IQR interquartile range, HR hazard ratio, 95% CI 95% confidence interval, FDR false discovery rate, Bolded markers have a p < 0.1

*Calculated on logarithm values of biomarker.

### Survival analysis of IL-8

**Figure 1:**
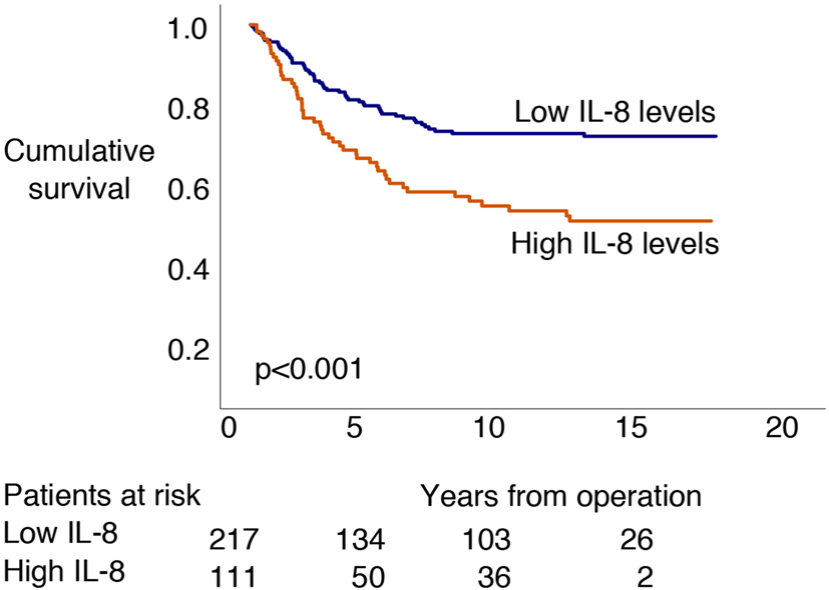
Disease-specific survival according to logarithm base 10 IL-8 levels based on the log-rank test (Kaplan–Meier). The cohort was dichotomized according to the maximum Youden values for IL-8.

**Figure 2:**
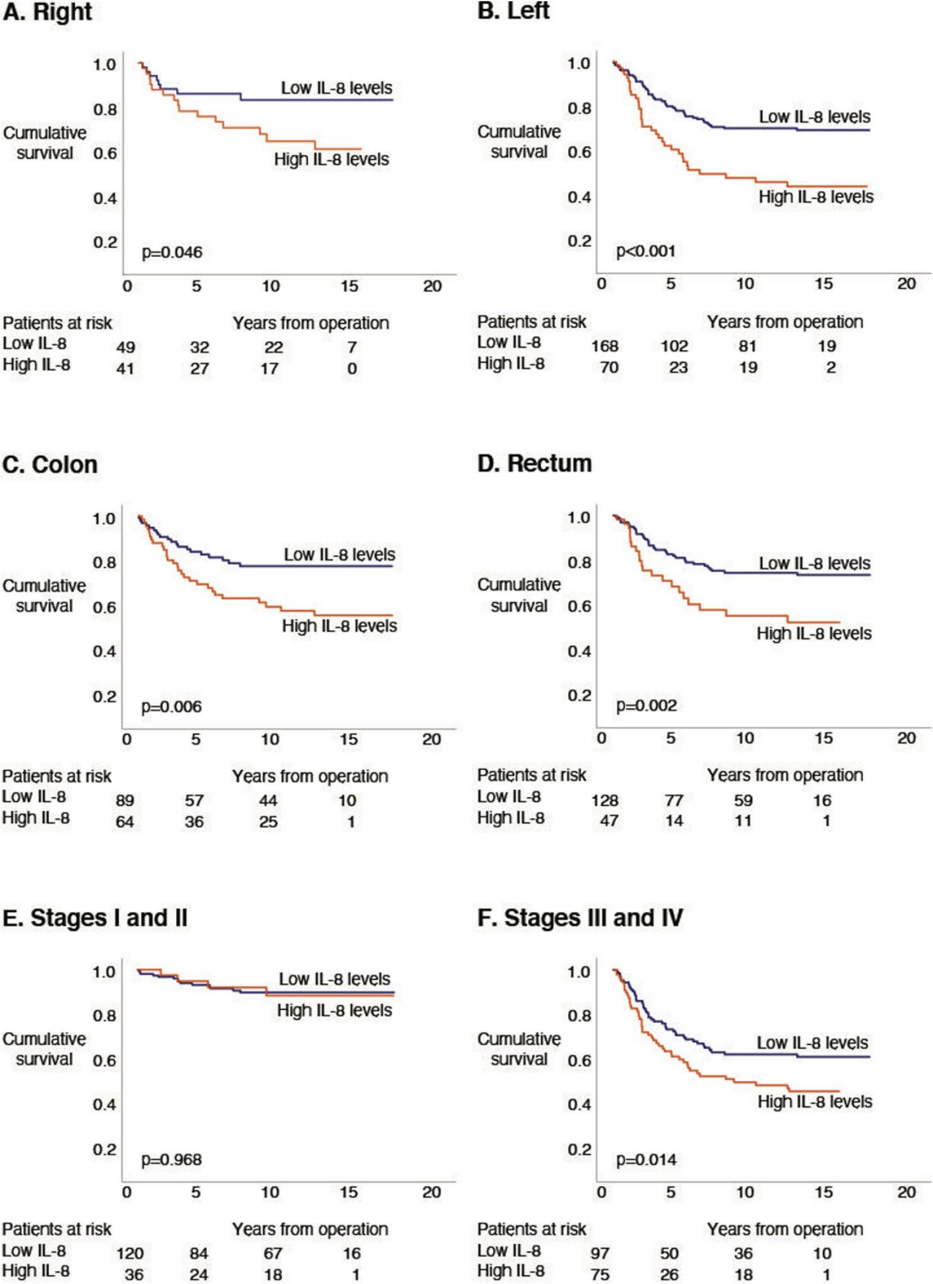
Disease-specific survival according to the log-rank test of logarithm base 10 IL-8 subgroup analyses (Kaplan–Meier). The cohort was dichotomized using the maximum Youden values as the cutoff for IL-8. (**A**) IL-8 levels for right-sided disease. (**B**) IL-8 levels for left-sided disease. (**C**) IL-8 levels for colon cancer. (**D**) IL-8 levels for rectum cancer. (**E**) IL-8 levels for stages I and II. (**F**) IL-8 levels for stages III and IV.

We dichotomized IL-8 levels using the maximum point of the Youden index for the Kaplan–Meier survival analyses (Fig. 1). For the subgroup Cox regression analyses, IL-8 levels were analyzed as a continuous variable (Table 2). We identified no significant difference in survival among patients with right-sided disease (HR 2.34; 95% CI 0.99–5.52; *p* = 0.052; Fig. 2A and Table 2). Patients with left-sided disease and high IL-8 levels exhibited a poor prognosis compared to patients with low IL-8 levels (HR 2.29; 95% CI 1.48–3.55; *p* < 0.001; Fig. 2B and Table 2). Overall, colon cancer patients with high IL-8 levels exhibited a poor prognosis compared to those with low IL-8 levels (HR 2.19; 95% CI 1.23–3.88; *p* = 0.008; Fig. 2C and Table 2). Additionally, patients with rectal cancer and high IL-8 levels exhibited a poor prognosis compared to rectal cancer patients with low IL-8 levels (HR 2.23; 95% CI 1.31–3.80; *p* = 0.003; Fig. 2D and Table 2). Among patients with stages I or II, IL-8 did not serve as a prognostic factor (HR 1.02; 95% CI 0.34–3.11; *p* = 0.968; Fig. 2E and Table 2), whereas patients with stages III or IV disease and a high IL-8 level exhibited a poor prognosis compared to stage III or IV patients with low IL-8 levels (HR 1.67; 95% CI 1.11–2.53; *p* = 0.015; Fig. 2F and Table 2).

**Table 2:** Association analysis and univariate analysis for logarithm 10 levels of IL-8 among colorectal cancer patients.

Association analyses (Mann–Whitney *U* or Kruskall–Wallis test)				Univariate hazard ratio for disease-specific survival^3^			
	Median value (pg/ml)	IQR	p value^1^		HR	95% CI	p value^4^
**Gender**							
Female	43	32–64	0.802		1.67	0.96–2.9	0.067
Male	46	33–60			2.39	1.42–4.02	**0.001**
**Age**							
≤ 67	46	35–63	0.221		1.86	1.05–3.28	**0.033**
> 67	42	32–59			2.37	1.42–3.95	**0.001**
**Stage**				**Stage groups**			
I	37	28–47	**< 0.001**^**2**^	I–II	1.02	0.34–3.11	0.968
II	43	35–57		III–IV	1.67	1.11–2.53	**0.015**
III	40	31–61					
IV	60	45–110					
**Histology**							
Non-mucinous	43	33–60	0.800		1.85	1.24–2.75	**0.003**
Mucinous	45	31–76			7.76	1.57–38.3	**0.012**
**Side**							
Right	49.46	37–68	**0.005**		2.34	0.99–5.52	0.052
Left	41.62	31–58			2.29	1.48–3.55	**< 0.001 **
**Location**							
Colon	49	35–66	**0.005**		2.19	1.23–3.88	**0.008**
Rectum	41	31–57			2.23	1.31–3.80	**0.003**

* IQR* interquartile range, *HR* hazard ratio, *95% CI* 95% confidence interval.

^1^p value for the Mann–Whitney U test.

^2^p value for the Kruskall-Wallis test.

^3^IL-8 dichotomized using the reciever operating curve's maximal Yonden value.

^4^p value for the log-rank test.

p-values 0.050 or less are bolded.

Furthermore, we completed a multivariate survival analysis using a Cox regression model for IL-8 with the background characteristics of age at diagnosis, tumor location, stage and gender. IL-8 served as an independent prognostic marker (HR 1.01; 95% CI 1.00–1.01; *p* = 0.012) along with stages III and IV and age at diagnosis.

### Association analysis

IL-8 levels were significantly lower in patients with left-sided disease when compared to patients with right-sided disease (Mann–Whitney U test, *p* = 0.005; Table 2). IL-8 levels were significantly lower among patients with colon cancer compared to rectal cancer (Mann–Whitney U test, *p* = 0.005; Table 2). Yet, IL-8 serum levels differed significantly between stages I, II, III and IV (Kruskall–Wallis test, *p* < 0.001; Table 2).

### Multivariate survival analysis

We developed the background model based on clinical and patient characteristics (gender, tumor location, stage classification, CEA levels and age). In doing so, we compared the study model created using the random forest survival techniques for this background model to identify potential candidate prognostic markers of CRC survival to study further. CEA was included in the background model since it is an established prognostic marker, often called the gold standard marker for CRC^12^. The integrated AUC (6–60 months) for the background model was 0.812. If patients were divided into high-risk and low-risk groups by optimizing the cut-off value for the linear predictor score of the background model using the maximal Youden index, the five-year survival for the low-risk group was 87.0% (95% CI 81.8–92.2%) and 47.0% (95% CI 38.3–55.8%) for the high-risk group.

**Figure 3:**
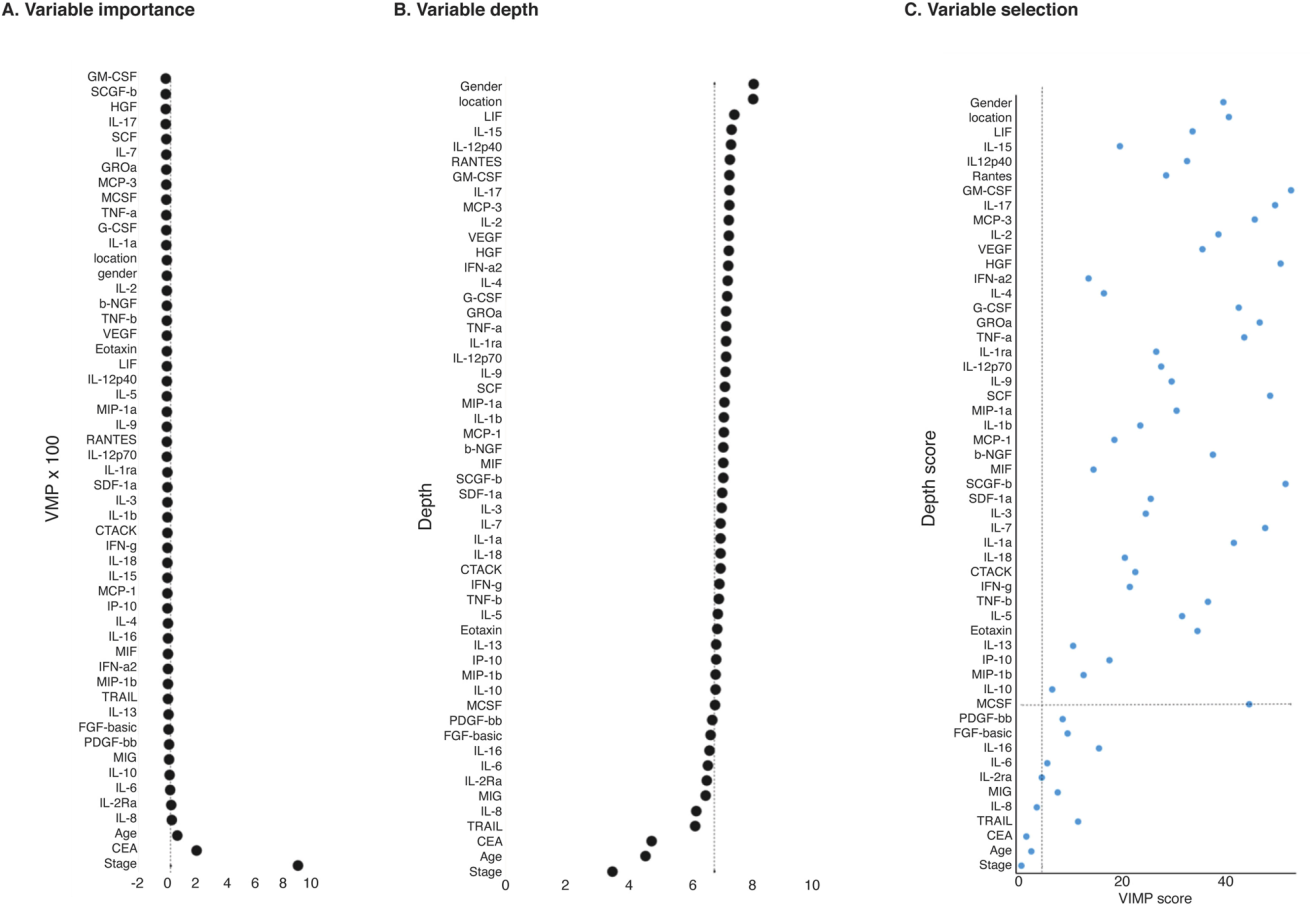
(**A**) List of variable importance compared to stepwise added background noise. Values greater than 0 indicate greater importance for a variable than the added background noise. (**B**) List of variables according to the mean value at which the variable has a division base in the random forest survival tree. A lower value indicates greater importance. (**C**) Variable selection based on the random survival forest method. Comparison of the value of importance (VIMP) score and minimal depth score. Dashed lines show the selected thresholds for the minimal depth (6.74) and VIMP (0.0025; mean value). Variables in the lower-left quadrant carry some importance in the random forest survival model. The most important parameters lie closest to the origin.

We used all of the biomarkers and variables applied to the background model for the random survival forest model. Given the small dataset, we used all patients for learning; therefore, only tentative results can be obtained from this analysis. Variable selection based on the value of the minimal depth above the threshold (6.74) and the value of importance above the threshold (0.025) yielded two tentative candidate CRC prognostic markers: IL-8 and IL-2Ra (Fig. 3). However, these thresholds are somewhat arbitrary, although the most prominent markers appear in the lower-left corner of Fig. 3. Another way to present the differences in survival according to the background model and the study model is by using the Kaplan–Meier curves (Supplementary Fig. 1). The integrated AUC for the random survival forest model was 0.943 (6–60 months). If patients were divided into high- and low-risk groups by optimizing the cut-off for the linear predictor score of the random survival forest model using the maximal Youden index, 5-year survival for the low-risk group reached 97.1% (95% CI 94.6–99.6%), falling to 25.3% (95% CI 17.0–33.6%) for the high-risk group.

## Discussion

We investigated 48 chemokines in 328 CRC patients, and developed a multivariate learning model, thereby improving the prognostic assessment of CRC patients. Specifically, we found that IL-8 served as a significant prognostic marker for CRC survival.

In our ELISA multiplex analysis of 48 cytokines, we found 6 biomarkers with *p* < 0.1 in the univariate analysis. These were IL-6, IL-8, IL-2Rα, MIF, CTACK and SDF-1α, with only IL-8 reaching *p* < 0.05 following FDR correction. IL-6 is a proinflammatory cytokine involved in tumor growth, invasion and metastasis, known to be elevated in CRC patients with a poor prognosis^13,14^. However, IL-6 did not emerge as a significant prognostic factor for CRC in our study. T-cells express IL-2Rα, which plays a role in early CRC development by suppressing T-cell activation^15,16^. IL-2Rα was also elevated in our study, suggesting it may play a role in the systematic inflammatory response in CRC, resulting in a worse prognosis^17^. In a study based on preoperative serum samples from 96 CRC patients, IL-2Rα served as a significant independent prognostic factor in CRC^18^.

Hypoxia tolerance represents one step in tumor development, and one hypoxia pathway gene known to overexpress in CRC tumors is *MIF*^19^. *MIF* activation also plays a role in chemotherapy resistance and participates in parallel intrinsic pathways in KRAS-driven CRC, promoting cell growth and proliferation^20^. We identified elevated MIF serum levels in our sample, also suggesting an increased protein expression. Yet, further research, such as immunohistochemistry and proximity ligand assays (PLAs), for example, are necessary in order to conclusively determine MIF’s role in CRC prognosis. Furthermore, we identified elevated CTACK levels, indicating that it plays a significant role in CRC prognosis. CTACK is a cutaneous T-cell attracting the C–C motif (two adjacent cysteines) chemokine participating in inflammatory and immunoregulatory processes. In addition, CTACK recruits T-cells to cutaneous sites and elevated levels accompany Epstein–Barr virus-induced mucosal carcinoma^21^. In a case–control study by Song et al. among 437 CRC patients and a random subcohort among 774 patients, CTACK carried no predictive value^22^. However, elevated levels of serum CTACK appeared in patients with hepatocellular carcinoma treated with and responding to radiofrequency ablation or transarterial chemoembolization^23^. The reason for this discrepancy between previous findings and ours remains unclear.

The serum levels of SDF-1α were elevated in patients with a poor prognosis in our multivariate model, indicating that it represents a significant prognostic factor in CRC. A previous study found that *SDF-1α* overexpression functioned as a significant prognostic marker in a cohort of 163 CRC patients carried out on formalin-fixated paraffin-embedded tissue samples; yet, no studies have examined the serum levels^24^.

We also found that high IL-8 levels associate with an impaired prognosis, locally advanced disease and metastatic disease. This agrees with a meta-analysis by Xia et al. among 1509 CRC patients, indicating that IL-8 represents a potent indicator for CRC progression^25^. Furthermore, IL-8 plays a role in cancer cell survival, proliferation and chemoresistance, and was further shown to play an active role in the CRC cell endothelial-to-mesenchymal transition (EMT)^26^. EMT is a critical developmental point for cancer cells, whereby epithelial cells undergo expression changes obtaining mesenchymal properties, thereby facilitating local invasion and representing a key point for adenocarcinoma metastasis^27^.

We succeeded in developing a refined and statistically advanced learning model with potent properties for clinical use. The integrated AUC for the random survival forest model of 0.943 (6–60 months) is, of course, quite good, since it uses all of the data. Because we could not use a test group, further refinements and validation are needed before we can make any definitive claims regarding its clinical role. Nevertheless, this model appears promising as a conventional multiplex or ELISA marker kit in CRC prognostics. Consensus regarding how to further develop and apply multivariate prognostic models to clinical practice remains unresolved^7^. The survival time difference increased in our learning model compared to the more general background model. We also found significant biomarkers in the multivariate model, yet these did not emerge as significant factors in the univariate analysis after FDR correction.

Our study’s limitation lies in the lack of detailed data on adjuvant and neoadjuvant radiation- and chemotherapies. We also chose not to create predictive models with C-reactive protein (CRP), since we focused instead on the chemokines. It remains unclear whether including CRP would alter these results. One weakness is the fact that the concentrations of different molecules in the serum samples may decrease during long-term storage, even at – 80 °C. This has not been tested in our samples, but the measurements were performed from sera thawed for the first time when assayed. In this study, we used serum for the multiplex measurements. We did not compare serum and plasma samples. Thus, it is possible that part of the molecules measured derive from granules from platelets and granulocytes in the serum sample.

## Conclusions

IL-8 represents a significant prognostic biomarker in colorectal cancer (CRC). Multivariate prognostic models remain promising and useful tools in the prognostics for CRC patients. Survival time analysis improved in our learning model. Further trials using our AI-based model are warranted in order to improve the prognostic stratification of CRC patients.

## Supplementary Information

Supplementary Figure 1. Disease-specific survival according to the log-rank test of the multivariate analyses. (A) Background model including age, gender, tumor location, stage classification and CEA. (B) Study model based on 48 biomarkers, age, gender, tumor location and stage classification.

Supplementary Table 1. Patient charasteristics in 328 colorectal cancer patients. Abbreviations: IQR = interquartile range; CEA = Carcinoembryonic antigen.

Supplementary Table 2. List of biomarkers used in the enzyme-linked immunosorbent assay.
