# Supplementary material for: A prognostic model for colorectal cancer based on CEA and a 48-multiplex serum biomarker panel
Source: Sci Rep. 2021 Feb 22;11:4287. doi: 10.1038/s41598-020-80785-1 (PMC7900104; doi:10.1038/s41598-020-80785-1)

A Prognostic Model for Colorectal Cancer based on CEA and a 48-multiplex Serum Biomarker Panel

Kajsa Björkman1, Sirpa Jalkanen2, Marko Salmi2, Harri Mustonen1, Tuomas Kaprio1, Henna Kekki3, Kim Pettersson3, Camilla Böckelman1,4* and Caj Haglund1,4*

1Research Programs Unit, Translational Cancer Medicine, University of Helsinki, Helsinki, Finland
2MediCity Research Laboratory and Institute of Biomedicine, University of Turku, Turku, Finland
3Department of Biochemistry, University of Turku, Turku, Finland
4Department of Surgery, University of Helsinki and Helsinki University Hospital, Helsinki, Finland
*Shared last authorship

Supplementary figure 1

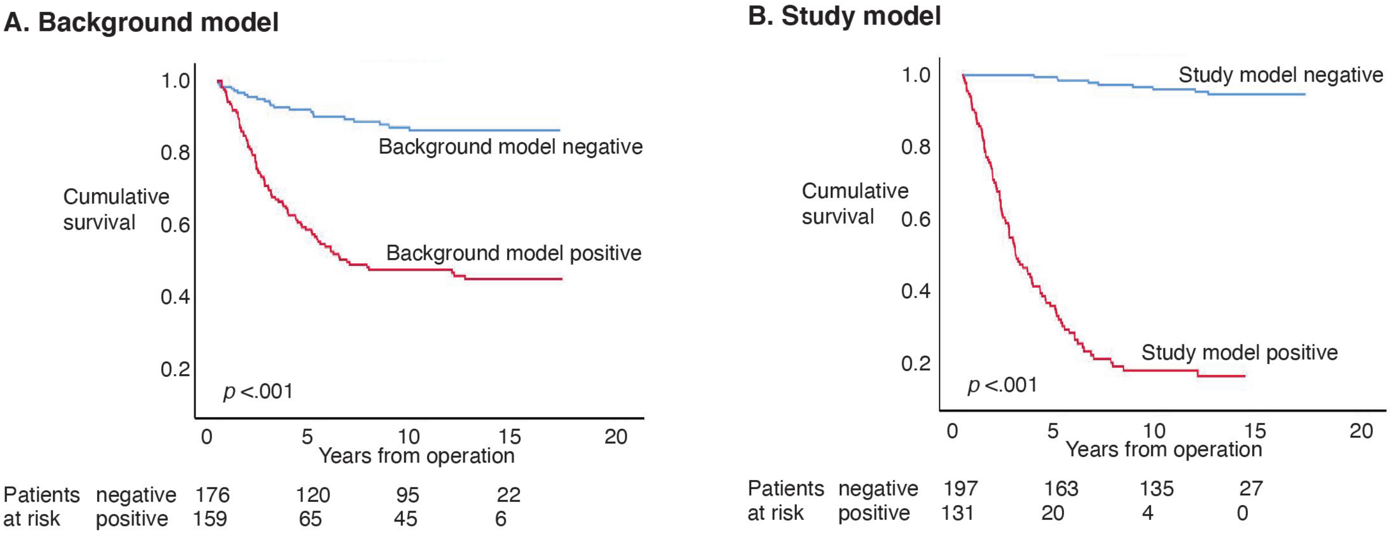

Supplement: Supplementary file 1 — Supplementary Figure 1. Disease-specific survival according to the log-rank test of the multivariate analyses. (A) Background model including age, gender, tumor location, stage classification and CEA. (B) Study model based on 48 biomarkers, age, gender, tumor location and stage classification. [file 41598_2020_80785_MOESM1_ESM.pdf]
